# Identification of Circulating Long Noncoding RNA Linc00152 as a Novel Biomarker for Diagnosis and Monitoring of Non-Small-Cell Lung Cancer

**DOI:** 10.1155/2017/7439698

**Published:** 2017-11-19

**Authors:** Nandi Li, Xiao Bo Feng, Qian Tan, Ping Luo, Wei Jing, Man Zhu, Chunzi Liang, Jiancheng Tu, Yong Ning

**Affiliations:** ^1^Department of Clinical Laboratory Medicine and Center for Gene Diagnosis, Zhongnan Hospital of Wuhan University, Wuhan, Hubei, China; ^2^Department of Anesthesiology, Zhongnan Hospital of Wuhan University, Wuhan, Hubei, China; ^3^School of Laboratory Medicine, Hubei University of Traditional Chinese Medicine, Wuhan, Hubei, China

## Abstract

**Objective:**

Long noncoding RNAs (lncRNAs) have been reported to play vital roles in non-small-cell lung cancer (NSCLC). Recently, long noncoding RNA Linc00152 has been reported to play important roles in various cancers. In this study, our aim was to investigate its expression pattern and clinical significance and further evaluate its diagnostic value for NSCLC.

**Methods:**

The levels of Linc00152 were detected in NSCLC tissues and plasma samples by quantitative real-time PCR (qRT-PCR). Receiver operating characteristic (ROC) curves were depicted to evaluate the diagnostic value.

**Results:**

We found that Linc00152 levels were upregulated in both NSCLC tissues and plasma samples. Plasma Linc00152 levels were significantly lower in postoperative samples than in preoperative samples. Besides, high Linc00152 expression was significantly correlated with tumor size (*r* = 0.293, *P* = 0.005) and tumor stage (*r* = 0.324, *P* = 0.011). The ROC curves indicated that plasma Linc00152 has high diagnostic accuracy for NSCLC, and the area under curve (AUC) for NSCLC versus healthy was 0.816 (95% CI: 0.757–0.875). Moreover, we found that the combination of Linc00152 and CEA could provide a more powerful diagnosis efficiency than Linc00152 or CEA alone (AUC = 0.881, 95% CI: 0.836–0.926).

**Conclusions:**

Plasma Linc00152 could serve as a promising biomarker for diagnosing and monitoring NSCLC.

## 1. Introduction

Lung cancer is the most common cancer worldwide. Cancer statistics reported that China had an estimated 4292,000 new cancer cases and 2814,000 cancer deaths in 2015, with lung cancer being the leading cause of cancer death [1]. Non-small-cell lung cancer (NSCLC) accounts for approximately 85% of lung cancer cases, which includes squamous cell carcinoma, adenocarcinoma, large cell carcinoma, and several other types [2]. Although some encouraging progresses in treatment for cancer have been achieved, the 5-year survival rate of NSCLC patients is still frustrated [3]. At present, the sensitivity of traditional biomarkers of NSCLC, such as CYFRA21, CEA, NSE, and CA19-9, is far from satisfaction. Therefore, it is urgent to find new noninvasive and sensitive biomarkers to complement and improve the current NSCLC screening methods.

Tumor cells could release substantial amounts of RNA into the peripheral blood and are present at sufficient levels for quantitative analyses [4]. Circulating microRNAs (miRNAs) have been shown to be stable and could serve as reliable biomarkers for cancers [5–7]. Recently, many studies have focused on long noncoding RNAs (lncRNAs) [8–10].

LncRNAs are a type of transcripts longer than 200 nucleotides in length, which are poorly conserved and not translated into proteins [11]. Multiple lines of study have demonstrated that lncRNAs play a vital role in many biological processes, especially in tumor biology [12]. It has been reported that circulating lncRNAs could reflect the pathological and physiological change of cancer patients, which attracted much attention on identification of circulating lncRNA as a biomarker for cancer patients [13, 14]. For instance, circulating lncRNA HULC could serve as a novel biomarker for gastric cancer [15]. However, there are still few studies regarding circulating lncRNAs for the diagnosis of NSCLC patients.

Linc00152, a novel lncRNA located at 2p11.2, has been demonstrated to be upregulated in gastric cancer [16, 17], hepatocellular carcinoma [18, 19], clear cell renal cell carcinoma [20], colon cancer [21], and gallbladder cancer [22, 23]. Moreover, recent studies indicated that Linc00152 could act as a circulating biomarker for esophageal squamous cell carcinoma and gastric cancer [24, 25]. However, the potential clinic value of Linc00152 in NSCLC has not been investigated. Therefore, in this study, we aimed to study the expression pattern and clinical significance of Linc00152 in NSCLC and then further investigate whether circulating Linc00152 could serve as a potential biomarker for NSCLC.

## 2. Material and Methods

### 2.1. Ethics Approval

All subjects gave their informed consent for inclusion before they participated in the study. The study was conducted in accordance with the Declaration of Helsinki, and the protocol was approved by the Ethics Committee of Zhongnan Hospital of Wuhan University (Ethical Approval number 2013059). All the experiments were conducted according to the institutional ethical guidelines.

### 2.2. Sample Collection

72 paired NSCLC tissues and corresponding adjacent normal tissues were collected from patients who underwent surgical resection without preoperative chemotherapy or radiotherapy from Zhongnan Hospital of Wuhan University between January 2015 and July 2016. All NSCLC tissues and corresponding adjacent normal tissues were stored at −80°C before use.

We collected whole blood samples from 100 NSCLC patients (including 24 patients whose tissues were collected), 100 healthy controls, and 48 benign lung disease patients. Benign lung disease patients included pulmonary tuberculosis (*n* = 14), pulmonary inflammatory pseudotumor (*n* = 8), pulmonary benign sarcoidosis (*n* = 12), and pneumonia (*n* = 14). The whole blood samples were obtained from NSCLC patients before any anticancer treatment such as surgery, radiotherapy, and chemotherapy. Then, we collected 47 paired postoperative whole blood samples after approximately one month. Plasma was separated from a whole blood sample by two-step centrifugation (2000*g* for 5 min at 4°C and 12000*g* for 5 min at 4°C) to exclude cellular nucleic acids.

All NSCLC patients were recruited based on the histopathology results. Tumor stage was determined according to the National Comprehensive Cancer Network (NCCN) criteria. We collected clinical information of participants from the clinical laboratory of Zhongnan Hospital of Wuhan University, including age, gender, pathology type, tumor size, tumor stage, lymph node metastasis, and tumor biomarkers.

### 2.3. RNA Extraction and Reverse Transcription

We used Trizol reagent (Invitrogen, CA, USA) to extract total RNA from tissues and used the blood sample total RNA rapid extraction kit (BioTeke, China) to extract total RNA from plasma according to manufacturer's instruction. Then, RNA was reverse transcribed to cDNA by using PrimeScript RT reagent kit (Takara, China) in 20 *μ*l solution containing 7 *μ*l of RNA extract. The reverse transcription procedure was as follows: 42°C for 2 min, 37°C for 15 min, and 85°C for 5 sec.

### 2.4. Real-Time PCR Analysis

Real-time PCR (RT-PCR) was performed on the Bio-Rad CFX96 (Bio-Rad, CA, USA) using the SYBR Premix Ex TaqTM II real-time PCR kit (Takara, China) in a 20 *μ*l reaction volume. The reaction mixtures were incubated at 95°C for 5 min and then 40 cycles of denaturation at 95°C for 30 sec, annealing at 63.4°C for 30 sec, and elongation at 72°C for 30 sec. Glyceraldehyde-3-phosphate dehydrogenase (GAPDH) was found to be stably expressed in plasma and was regarded as an ideal internal control. In this study, GAPDH was selected as an internal reference gene to normalize the results of RT-PCR. We found that GAPDH levels were stable in tissues and plasma (*P* = 0.340). The sequence of primers were as follows: Linc00152 (forward: 5′-GTGATGTCCCCAGTGATCCA-3′ and reverse: 5′-TATTCGAGGGATGCAGACGG-3′); GAPDH (forward: 5′-GGTCTCCTCTGACTTCAACA-3′ and reverse: 5′-GTGAGGGTCTCTCT-CTTCCT-3′). The cycle threshold (Ct) is defined as the number of cycles required for the fluorescent signal to cross the threshold in RT-PCR. The relative expression was calculated by using the 2^−ΔCt^, and ΔCt = Ct_target gene_ − Ct_GAPDH_. All samples were analyzed in duplicate with no-template controls included.

### 2.5. Statistical Analysis

All statistical analyses were performed using SPSS software package version 19.0 (SPSS, Chicago, IL, USA). The figures were drawn by GraphPad Prism 5.0 software (GraphPad Software, La Jolla, CA, USA). The paired-sample *t* test was used to compare differences of Linc00152 expression in paired tissues and paired plasma samples before and after surgery. The independent two-tailed test was used to compare the levels of Linc00152 between NSCLC plasma samples and healthy control plasma samples. The two-side chi-square test was used to analyze the association between the expression level of Linc00152 and clinicopathological features. Correlations between the expression level of plasma and tissue were analyzed using the Spearman correlation. Receiver operating characteristic (ROC) curves and the area under the curve (AUC) were applied to evaluate the diagnostic value. *P* < 0.05 was considered to be statistically significant.

## 3. Results

### 3.1. Accuracy of RT-PCR Methods

Within-batch and between-batch coefficient of variation (CV) of Ct value were used to evaluate the repeatability and precision of the RT-PCR. The within-batch CV of Linc00152 and GAPDH for NSCLC patients and healthy controls were all <5%, and the between-batch CV were all <5% (Table 1).

### 3.2. Linc00152 Was Significantly Upregulated in NSCLC Tissues

**Figure 1:**
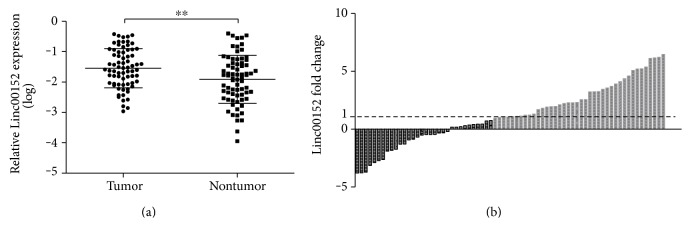
(a) The expression levels of Linc00152 in NSCLC tissues and adjacent normal tissues, ^∗∗^*P* < 0.001; (b) Waterfall plot showed Linc00152 was increased by at least twofold in 55.6% (39/72) of NSCLC tissues. The gray areas represent that linc00152 was upregulated by at least twofold. Fold change was represented by 2^−ΔΔCt^, and −ΔΔCt = −[(Ct_Linc00152_ − Ct_GAPDH_) of NSCLC − (Ct_Linc00152_ − Ct_GAPDH_) of normal].

Aiming to investigate the expression pattern of Linc00152 in NSCLC, Linc00152 expression levels were measured in 72 paired NSCLC tissues and corresponding adjacent normal tissues by RT-PCR. As shown in Figure 1(a), Linc00152 expression levels were remarkably upregulated in NSCLC tissues compared to adjacent normal tissues (*P* < 0.001). Besides, waterfall plot showed that Linc00152 was upregulated in 70.8% (61/72) of NSCLC tissues and was increased by at least twofold in 55.6% (39/72) of NSCLC tissues (Figure 1(b)). These results indicated that Linc00152 may exert an oncogenic role in NSCLC.

NSCLC patients were divided into the low expression group (*n* = 36) and high expression group (*n* = 36) according to the median expression level of Linc00152. The correlation between Linc00152 and clinicopathological features of NSCLC patients was analyzed. The univariate analysis revealed that high Linc00152 expression was significantly correlated with tumor size (*P* = 0.005) and tumor stage (*P* = 0.011, Table 2). However, the multivariate regression analysis showed that Linc00152 expression was only significantly associated with tumor size (*P* = 0.005, Table 2).

### 3.3. Analysis of Stability of Linc00152 in Plasma

**Figure 2:**
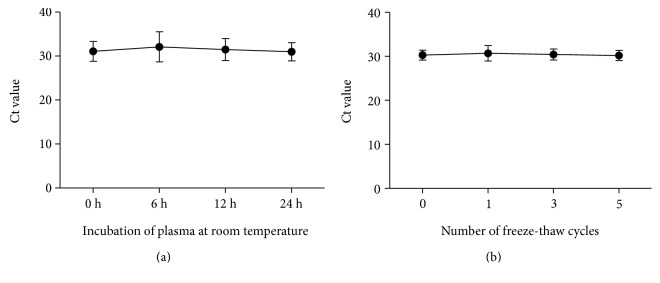
The stability of Linc00152 plasma in harsh environments. Incubation of plasma at room temperature (a) and the freeze-thawing processes (b).

To analysis the stability of Linc00152 in plasma, five healthy plasma samples were divided into four parts and left under harsh conditions such as incubation at room temperature for 0, 6, 12, and 24 hours or repeated freeze-thaw cycles. No obvious alterations were observed in plasma Linc00152 levels under both of the above conditions (*P* > 0.05, Figures 2(a) and 2(b)). These results demonstrated that Linc00152 remained stable in plasma and whole blood, which laid the foundation for detecting Linc00152 levels in plasma.

### 3.4. Increased Linc00152 in Plasma of NSCLC Patients

**Figure 3:**
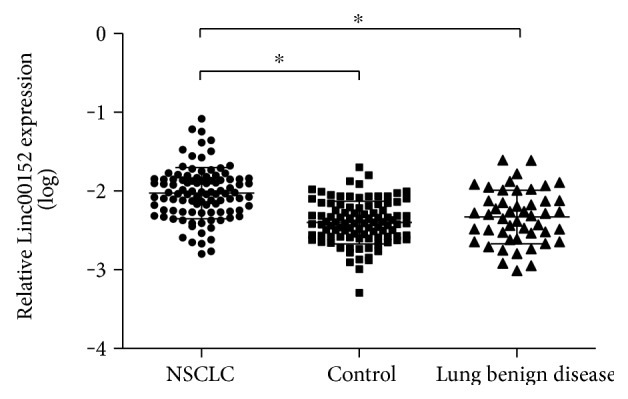
The levels of plasma Linc00152 in NSCLC patients (*n* = 100), healthy controls (*n* = 100), and benign lung disease (*n* = 48). ^∗^*P* < 0.05.

Previous results showed that Linc00152 levels were significantly upregulated in NSCLC tissues and remained stable in whole blood and plasma. Thus, we further measured plasma Linc00152 levels in 100 NSCLC patients and 100 healthy controls. As shown in Figure 3, plasma Linc00152 levels were remarkably higher in NSCLC patients than in benign lung disease patients and healthy controls (*P* < 0.001). However, there was no difference between benign lung disease and healthy controls (*P* = 0.169). The clinical information of studied subjects was summarized in Supplementary Table 1 available online at https://doi.org/10.1155/2017/7439698. There was a significant difference in concentrations of red blood cells (RBC) and hemoglobin (HGB) between the two groups. However, no difference was observed in age, gender, white blood cells (WBC), and platelets (PLT) between the two groups. We examined the associations of plasma Linc00152 levels with NSCLC traditional tumor biomarkers. Plasma Linc00152 levels had a statistical relevance with CEA (*P* < 0.001, Supplementary Table 2).

### 3.5. Diagnostic Value of Plasma Linc00152 for NSCLC

**Figure 4:**
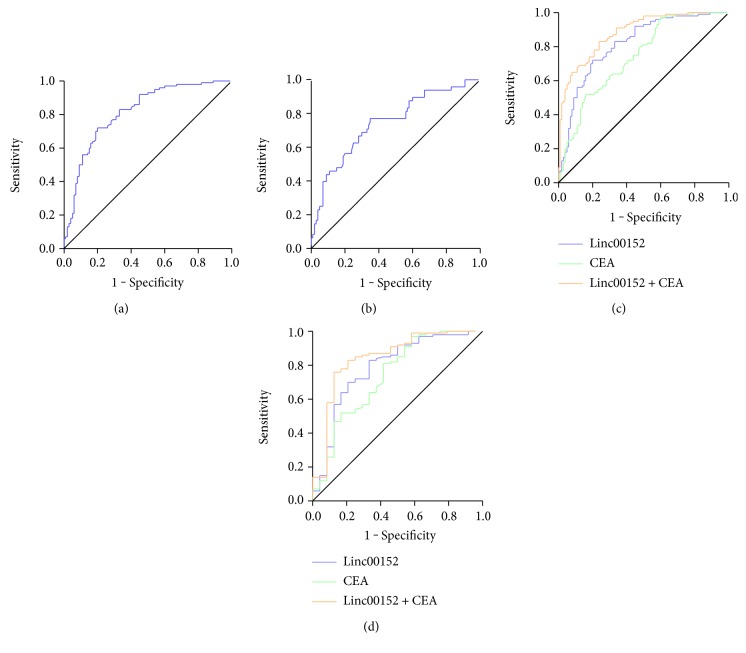
(a-b) ROC curve for distinguishing NSCLC patients from healthy controls (a) or benign lung disease patients (b). (c-d) ROC curves to compare the diagnostic performance of Linc00152, CEA, and a combination of Linc00152 and CEA to discriminate all NSCLC patients (c) or early stage NSCLC patients (d) from healthy controls.

To further assess the potentiality of clinical application of plasma Linc00152, ROC curves were constructed on data from 100 NSCLC patients and 100 healthy controls. The AUC of Linc00152 was 0.816 (95% CI: 0.757–0.875, Figure 4(a)) for distinguishing NSCLC patients from healthy controls, and the optimal sensitivity and specificity were 80% and 72%, respectively (Table 3). Meanwhile, Linc00152 also has a good diagnostic efficiency for differentiating NSCLC patients from benign lung disease (AUC = 0.742, 95% CI: 0.656–0.828, Figure 4(b)).

Our results showed that Linc00152 has an association with CEA; thus, we combined Linc00152 with CEA in diagnosing NSCLC. The AUC of CEA was 0.741 (95% CI: 0.673–0.809), which was lower than Linc00152. We found that the combination of Linc00152 and CEA could greatly improve the diagnostic efficiency for diagnosing NSCLC (AUC = 0.881, 95% CI: 0.836–0.926, Figure 4(c)).

Furthermore, we further evaluated the diagnostic value of plasma Linc00152 levels for early stage NSCLC patients (stage I). The AUC of Linc00152 was 0.786 (95% CI: 0.674–0.899), which was higher than that of CEA (AUC = 0.738, 95% CI: 0.616–0.861). When we combined Linc00152 and CEA to diagnose stage I NSCLC patients, the diagnostic value was significantly improved (AUC = 0.844, 95% CI: 0.744–0.944, Figure 4(d)).

### 3.6. Evaluation of the Use of Linc00152 for Monitoring Tumor Dynamics in NSCLC Patients

**Figure 5:**
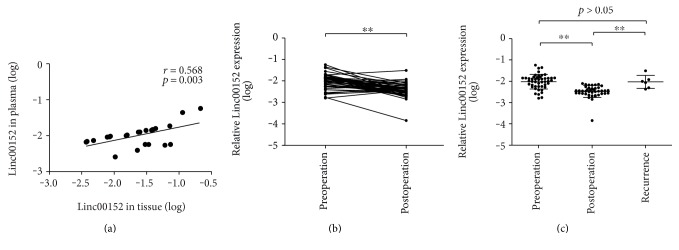
Dynamic monitoring of plasma Linc00152 levels in NSCLC patients. (a) Correlation of Linc00152 expression levels in tumor and plasma samples. (b) Comparison of plasma Linc00152 levels between pre- and postoperative samples. (c) Scatter plots of plasma Linc00152 levels from preoperative, postoperative, and recurrent patients, ^∗∗^*P* < 0.001.

We carried out two experiments to investigate whether plasma Linc00152 levels could monitor NSCLC dynamics. Firstly, RT-PCR was used to measure the expression in 24 paired NSCLC tumor tissues and plasma samples which were from the same individuals. The correlation of Linc00152 levels between the two groups was analyzed. Linc00152 levels has a significant correlation between tissue and plasma (*r* = 0.490, *P* = 0.015, Figure 5(a)).

Then, Linc00152 levels were analyzed in 47 paired pre- and postoperative plasma samples (after one month) from NSCLC patients. The results showed that plasma Linc00152 levels were significantly lower in postoperative samples than in preoperative samples (*P* < 0.001, Figure 5(b)). However, compared to NSCLC patients without recurrence, plasma Linc00152 levels of patients with recurrence were sharply increased (*P* < 0.05, Figure 5(c)). Based on the above results, we could presume that plasma Linc00152 levels might reflect tumor dynamics.

## 4. Discussion

With the development of microarray and massively parallel sequencing technology, accumulating studies indicated lncRNAs could serve as oncogenes or tumor suppressors during carcinogenesis [26]. Recently, several circulating lncRNAs have been characterized as potential tumor markers for cancers. However, there are still few studies reported circulating lncRNAs in the diagnosis of NSCLC patients. In this study, we set out to explore the expression pattern and clinical significance of Linc00152 in NSCLC and then further investigate whether Linc00152 could serve as a potential biomarker for NSCLC.

First, we confirmed the repeatability and precision of the qRT-PCR method by the intra-assay CV and interassay CV. Then, we investigated the expression and clinical significance of Linc00152 in NSCLC. We found that Linc00152 expression levels were higher in NSCLC tissues than in paired adjacent normal tissues and its levels correlated with tumor size. Our results were in accordance with the previous studies in other cancers [17, 18, 25], which suggested that Linc00152 may play an oncogenic role in NSCLC.

Circulating lncRNAs were thought to be unstable due to the presence of RNase [27]. Thus, we next sought to analyze the stability of circulating Linc00152. Our results suggested that Linc00152 levels remained stable in plasma, which was consistent with the results in gastric cancer [15]. One possible explanation was that circulating lncRNAs were packaged in exosomes to avoid the decomposition of RNase [25], and another possible explanation was that circulating lncRNAs could be modified in some ways, including methylation, adenylation, and uridylation which make them resistant to RNase digestion [28]. In a word, these results demonstrated that Linc00152 remained stable in plasma and whole blood, which could lay the foundation for evaluating plasma Linc00152 as a biomarker for NSCLC patients.

Then, we further found that plasma Linc00152 levels were remarkably higher in NSCLC patients than in benign lung disease patients and healthy controls. Linc00152 could provide a high diagnostic performance for distinguishing NSCLC patients from benign lung disease and healthy controls. Its AUC and sensitivity were higher than CEA, which is one of the most commonly used lung cancer biomarkers [29]. These results suggested that Linc00152 could serve as a promising biomarker for NSCLC diagnosis. Moreover, the combination of Linc00152 and CEA could provide a more accurate diagnosis than Linc00152 or CEA alone. Henschke et al. reported the patients with early stage lung cancer who underwent resection within one month after diagnosis had a significantly improved 10-year survival rate [30]. Thus, it is important to diagnose NSCLC patients at early stage for improving the survival rate of NSCLC patients. We found that Linc00152 could well distinguish NSCLC patients with early stage (AUC = 0.786, 95% CI: 0.734–0.873) from healthy people, and the combination of Linc00152 and CEA provided a more powerful diagnosis performance, which could substantially benefit to the treatment and prognosis of NSCLC.

Finally, we carried out two analyses to validate whether plasma Linc00152 levels could monitor NSCLC dynamics. Firstly, the correlation of Linc00152 levels between plasma and tissue samples was analyzed. Plasma Linc00152 levels had a significant association with tumor tissue. Secondly, Linc00152 levels were analyzed in 47 paired pre- and postoperative plasma samples (after one month) from NSCLC patients. Plasma Linc00152 levels were significantly decreased after operation. However, compared to NSCLC patients without recurrence, plasma Linc00152 levels of patients with recurrence were sharply increased. The possible explanation was that circulating lncRNAs were mainly derived from tumor cells [31], and it would presumably be back to normal after the removal of tumor, while recurrence of tumor will increase the supply to cause its abnormal raising. These results demonstrated that plasma Linc00152 levels could reflect tumor dynamics and monitor the status of NSCLC patients after operation.

In conclusion, this study investigated Linc00152 in NSCLC patients for the first time. We found that Linc00152 expression levels were upregulated in NSCLC tissues and correlated with tumor size. Moreover, we demonstrated that plasma Linc00152 could serve as a promising biomarker for diagnosing and monitoring NSCLC patients.

## Supplementary Material

Supplementary Table 1. PRISMA checklist for meta-analysis. Supplementary Table 2. Newcastle-Ottawa Assessment Scale scores for studies included in meta-analysis.

## Figures and Tables

**Table 1:** Within-batch and between-batch coefficient of variation (CV) of Linc00152 and GAPDH in the two groups.

	Group	Linc00152	GAPDH
Within-batch CV (%)	NSCLC	0.99	1.90
	Healthy control	1.74	1.60
Between-batch CV (%)	NSCLC	1.40	1.54
	Healthy control	1.88	1.98

NSCLC: non-small-cell lung cancer.

**Table 2:** Correlation between clinicopathological features and Linc00152 expression in NSCLC patients.

Characteristics	Numbers	High expression *n* = 36	Low expression *n* = 36	Univariate analysis	Multivariate analysis
Gender				0.276	0.958
Male	54	7	11		
Female	18	29	25		
Age (years)				0.634	0.155
<60	36	19	17		
≥60	36	17	19		
Histology subtype				0.336	0.268
ADC	34	14	20		
SCC	32	19	13		
Other	6	3	3		
Tumor size				**0.005** ^**∗**^	**0.035** ^**∗**^
≤3 cm	16	3	13		
>3 cm	56	33	23		
Tumor stage				**0.011** ^**∗**^	0.717
I/II	50	20	30		
III/IV	22	16	6		
Lymph node metastasis				0.125	0.420
No	50	14	8		
Yes	22	22	28		

SCC: squamous cell carcinoma; ADC: adenocarcinoma; ^∗^*P* < 0.05 was considered significant.

**Table 3:** Use of Linc00152 and CEA levels to distinguish NSCLC patients from healthy control.

	SEN	SPE	AC	PPV	NPV
Linc00152	80.0%	72.0%	76.0%	74.1%	78.3%
CEA	44.0%	86.0%	65.0%	75.9%	60.6%
Linc00152 + CEA	76.0%	83.0%	79.5%	81.7%	77.6%

SEN: sensitivity; SPE: specificity; AC: diagnostic accuracy; PPV: positive predictive value; NPV: negative predictive value.
